# Adaptations in Mitochondrial Enzymatic Activity Occurs Independent of Genomic Dosage in Response to Aerobic Exercise Training and Deconditioning in Human Skeletal Muscle

**DOI:** 10.3390/cells8030237

**Published:** 2019-03-12

**Authors:** Andreas M. Fritzen, Frank B. Thøgersen, Kasper Thybo, Christoffer R. Vissing, Thomas O. Krag, Cristina Ruiz-Ruiz, Lotte Risom, Flemming Wibrand, Louise D. Høeg, Bente Kiens, Morten Duno, John Vissing, Tina D. Jeppesen

**Affiliations:** 1 Copenhagen Neuromuscular Center, Section 3342, Rigshospitalet, University of Copenhagen, 2100 Copenhagen, Denmark; frank.thogersen@gmail.com (F.B.T.); kasperthy@gmail.com (K.T.); christoffervi@gmail.com (C.R.V.); thomas.krag@regionh.dk (T.O.K.); cruizruiz8@gmail.com (C.R.-R.); john.vissing@regionh.dk (J.V.); 2 Section of Molecular Physiology, Department of Nutrition, Exercise and Sports, Faculty of Science, University of Copenhagen, 2100 Copenhagen, Denmark; frkdalgas@hotmail.com (L.D.H.); bkiens@nexs.ku.dk (B.K.); 3 Department of Neurology, Rigshospitalet, University of Copenhagen, 2100 Copenhagen, Denmark; 4 Department of Clinical Genetics, Rigshospitalet, University of Copenhagen, 2100 Copenhagen, Denmark; lotte.risom@regionh.dk (L.R.); flemming.wibrand@regionh.dk (F.W.); morten.dunoe@regionh.dk (M.D.)

**Keywords:** mtDNA, mitochondria, skeletal muscle, exercise training, mitochondrial biogenesis

## Abstract

Mitochondrial DNA (mtDNA) replication is thought to be an integral part of exercise-training-induced mitochondrial adaptations. Thus, mtDNA level is often used as an index of mitochondrial adaptations in training studies. We investigated the hypothesis that endurance exercise training-induced mitochondrial enzymatic changes are independent of genomic dosage by studying mtDNA content in skeletal muscle in response to six weeks of knee-extensor exercise training followed by four weeks of deconditioning in one leg, comparing results to the contralateral untrained leg, in 10 healthy, untrained male volunteers. Findings were compared to citrate synthase activity, mitochondrial complex activities, and content of mitochondrial membrane markers (porin and cardiolipin). One-legged knee-extensor exercise increased endurance performance by 120%, which was accompanied by increases in power output and peak oxygen uptake of 49% and 33%, respectively (*p* < 0.01). Citrate synthase and mitochondrial respiratory chain complex I–IV activities were increased by 51% and 46–61%, respectively, in the trained leg (*p* < 0.001). Despite a substantial training-induced increase in mitochondrial activity of TCA and ETC enzymes, there was no change in mtDNA and mitochondrial inner and outer membrane markers (i.e., cardiolipin and porin). Conversely, deconditioning reduced endurance capacity by 41%, muscle citrate synthase activity by 32%, and mitochondrial complex I–IV activities by 29–36% (*p* < 0.05), without any change in mtDNA and porin and cardiolipin content in the previously trained leg. The findings demonstrate that the adaptations in mitochondrial enzymatic activity after aerobic endurance exercise training and the opposite effects of deconditioning are independent of changes in the number of mitochondrial genomes, and likely relate to changes in the rate of transcription of mtDNA.

## 1. Introduction

Mitochondrial content, volume, and enzyme activities are highly adaptable to changes in oxidative demand. Mitochondrial DNA (mtDNA) copy number is thought to change proportionally to these changes [1], and therefore mtDNA content is often used as a marker of mitochondrial content in skeletal muscle both in healthy humans [2,3,4], as well as in patients with type 2 diabetes [5,6,7,8], kidney disease [9], and obstructive pulmonary disease [10]. This notion has been substantiated by findings of a higher content of mtDNA in skeletal muscle of endurance-trained subjects compared to sedentary subjects [11,12], and increases in mtDNA content after 2–24 weeks of endurance exercise training in skeletal muscle in rodents [13,14,15] and humans [9,16,17,18,19]. Thus, findings of proportionality between mtDNA content and oxidative capacity in skeletal muscle have prompted the “gene dosage theory”: that mtDNA replication is an integral mechanism for exercise training-induced mitochondrial adaptations in enzyme activity and function [20]. However, findings of unchanged mtDNA levels, despite substantial changes in other biomarkers of mitochondrial function and content after endurance training [21,22,23,24] and in weight loss programs with combined diet and exercise training [2,3,4], have questioned this theory.

The aim of the present study was to examine the hypothesis that endurance-training- and deconditioning-induced changes in mitochondrial enzyme activity and function occur independently of genomic dosage in skeletal muscle. In order to investigate this, we conducted a study where mtDNA content was investigated in response to a well-controlled intervention, where subjects performed six weeks endurance training and subsequent four weeks deconditioning in one leg, keeping the other leg available for an interpersonal control.

## 2. Materials and Methods

### 2.1. Ethics

All participants gave their informed consent before participating in the study. The study was approved by the Ethics Committee of Copenhagen and Frederiksberg communities (KF 01-205/00) and performed in accordance with the Declaration of Helsinki.

### 2.2. Subjects and Initial Tests

Power calculations of sample size prior to initiation of the study based on previous mtDNA data variation and a desired treatment effect size of 20% estimated that a minimum of seven subjects were needed for adequate study power. In total, 13 healthy, sedentary male subjects were included in the study (Table 1). They were 22.9 ± 2.7 years old, had a body mass index (BMI) of 22 ± 4.0 kg/m^2^, and did not perform moderate-intensity training for more than one hour per week or more than four hours of weekly low-intensity bicycling. Of the 13 subjects, 10 completed the study. Three subjects were excluded: in two cases, due to noncompliant behaviour during the training period, and in one case, due to anxiety concerning the invasive methods of the study. To evaluate the training status of the participants, subjects initially completed an incremental cycle ergometer (Tunturi T6 plus Alpha 200, Almere, The Netherlands) test to exhaustion to determine whole-body maximal oxygen uptake (VO_2max_). All subjects followed the same test protocol, which included 5 min of exercise at 70 watts workload with subsequent increments of 30 watts added every minute until cessation of exercise due to volitional fatigue. Patients were instructed to keep a cadence of 80 rounds per minute (RPM) throughout the test. Additionally, body composition was determined by dual-energy X-ray absorptiometry scan (GE Medical Systems Lunar Prodigy, Chicago, IL, USA). Prior to the first test day, subjects were also familiarized with the knee-extensor exercise model [25].

### 2.3. Experimental Days

**Figure 1:**
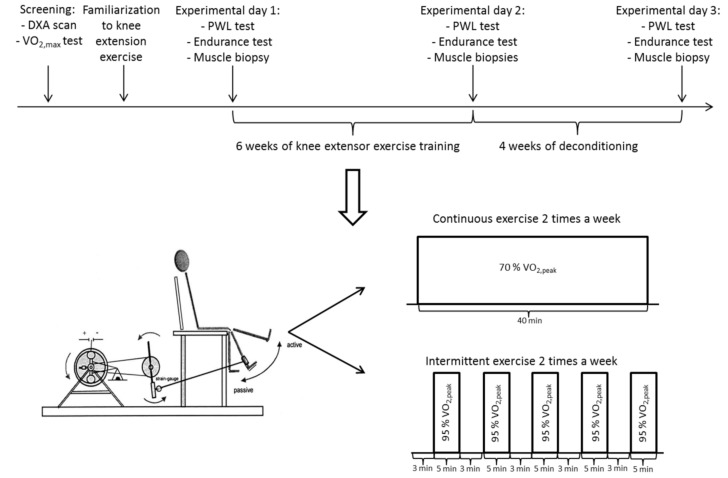
Schematic overview of the screening, familiarization, and experimental days prior and after the six-week one-legged knee-extensor exercise training intervention and after the four-week deconditioning period. In addition, the training intervention containing two continuous and two intermittent exercise bouts per week is depicted. DEXA, dual-energy X-ray absorptiometry; PWL, peak work load; VO_2,max_, maximal oxygen uptake during whole-body bicycle exercise; VO_2,peak_, peak oxygen uptake during one-legged knee-extensor exercise. Inspired by Andersen et al. [25].

On experimental days (Figure 1), subjects performed an incremental, one-legged, knee-extensor exercise test to determine both peak power output (peak work load (PWL)) for the knee extensors during exercise and peak oxygen uptake during knee-extensor exercise (VO_2,peak_). After a short active warm up without any resistance, increments of 5 watts per minute were added until volitional exhaustion. Oxygen uptake was obtained through measurements of breath-by-breath pulmonary gas exchange analysed by indirect calorimetry using an open-circuit online respirometer (Cosmed, Quark B2, Pavona, Italy). PWL was the maximal power output achieved and sustained for at least one minute during the incremental test. After 30 min of supine rest, a knee-extensor endurance test to exhaustion was performed. The initial load during the first 30 min of the endurance test was 80% of PWL generated at the incremental test. If achieved, exercise load was increased to 90% of PWL for 10 min. If achieved, further increments of 5% of PWL were added every 5 min until volitional exhaustion. Time to exhaustion was measured as the time-dependent capacity to sustain exercise at the workloads determined from the pretraining incremental test.

### 2.4. Training and Deconditioning

The subjects trained one leg on a knee-extensor model [25] four times weekly for six weeks under supervision at the laboratory (Figure 1). Every training session consisted of 40 min of exercise and changed between high-intensity intermittent exercise (first and third weekly session) and moderate-high continuous exercise (second and fourth weekly session). Intermittent exercise consisted of five periods of 5 min at a resistance corresponding to 95–100% of VO_2,peak_ followed by 3 min of active recovery at minimal resistance. In contrast, continuous exercise consisted of 40 min at 70% of VO_2,peak_. In both cases, the subjects were vocally encouraged at a standardized level to keep a steady cadence of 60 RPM at all times during training. Training load was adjusted during the training intervention to keep the relative intensity of training constant as measured by VO_2,peak_ during the maximal exercise in the intermittent exercise sessions. At the end of the six-week training period, subjects were asked to refrain from any unnecessary physical activity for a period of four weeks (deconditioning).

### 2.5. Muscle Biopsy

Before the training intervention, a biopsy was obtained from the vastus lateralis muscle after the endurance test from the trained leg. The biopsy was obtained under local anesthesia (lidocain 10 mg/mL) using a percutaneous Bergström needle. Another biopsy was obtained after the training period (three days after the last exercise training bout) and after the deconditioning period. A single biopsy was also obtained from the control, non-exercise-trained leg on the post-training test day. Tissue for histological analysis was arranged according to fiber direction and transferred into liquid-nitrogen-cooled isopentane (2-methylbutane 99.5%), while tissue for genetic and enzymatic analyses was snap-frozen in liquid nitrogen and stored at −80 °C for later analysis.

### 2.6. Quantitative PCR to Determine mtDNA Copy Number per Diploid Nuclear Genome

MtDNA copy number quantification was performed by qPCR using TaqMan probes (Applied Biosystems, Waltham, MA, USA). For the matter, two standard curves were amplified in all the separate runs, one built with growing concentrations of total human DNA and the other amplifying a fragment of mtDNA (chrM: 3221–3360) cloned in a pCR^TM^4Blunt-TOPO^®^ vector (Life Technologies, Waltham, MA, USA). For nDNA quantification, a commercial kit for copy number quantification amplifying the gene of Ribonuclease P (*RNase P*) was used (Applied Biosystems), following manufacturer recommendations. For mtDNA quantification, custom-made primers and probe were obtained from Applied Biosystems (Fw: ACAGGGTTTGTTAAGATGGC, Rv: TGCCATTGCGATTAGAATGG, FAM^®^ dye-labeled MGB^TM^ probe: AGAGCCCGGTAATCGCA). PCR reaction mixture for the amplification of *RNase P* in the standard curve and samples contained 10 μL of TaqMan Universal PCR MasterMix (Applied Biosystems), 1 μL of RNase P Expression Assay (Applied Biosystems), and 100 ng of DNA in a final volume of 20 μL. PCR reaction mixture for the amplification of mtDNA in the standard curve and samples contained 10 μL of TaqMan Universal PCR MasterMix (Applied Biosystems), 500 nM of each primer, 300 nM of the TaqMan probe, and 100 ng of DNA in a final volume of 20 μL. Real-time PCR conditions were 10 min at 95 °C, followed by 40 cycles of denaturation at 95 °C for 15 seconds and annealing and extension at 60 °C for 1 min. The fluorescent signals were recorded and analyzed during PCR in a CFX96^TM^ Real-Time System Thermal Cycler (Bio-Rad, Hercules, CA, USA) using the CFX Manager^TM^ Software version 3.1 by Bio-Rad. All samples and controls were run in duplicates. The relative mtDNA copy number was calculated as the ratio of the number of each of them. Knowing the size and weight of the amplified molecules, it was possible to determine the number of molecules of each DNA in the sample. Furthermore, the reproducibility was tested by measurement of mtDNA content relative to nDNA in another laboratory (data not shown) using a second primer set targeting coding regions.

### 2.7. Enzyme Activities

Enzyme activities of CS and respiratory chain complexes I–IV were measured in postnuclear supernatant of 30 mg of frozen muscle at 37 °C in a spectrophotometer (Varian Cary 100 Bio, Agilent, Santa Clara, CA, USA) as previously described [26]. Protein assessment was performed in a separate analysis and enzyme activity (nm substrate catalyzed) per minute was expressed relative to protein (mg) or muscle content.

### 2.8. Cardiolipin

Cardiolipin content was measured in freeze-dried and dissected skeletal muscle tissue. Lipids were initially extracted by a Folch extraction in chloroform-methanol (2:1, *v*/*v*). Then, phospholipids were separated by thin-layer chromatography, in which lipid extracts were spotted on silica gel plates (0.22 mm, kieselgel 60; Merck, Darmstadt, Germany) and developed in chloroform, methanol, acetic acid, and water (50:50:5:5, *v*/*v*/*v*/*v*). Then, the plates were dried under nitrogen. Standards of cardiolipin (Avanti Polar Lipids, Alabaster, AL, USA) were run along with the samples. The plates were immersed in a 10% (*w*/*v*) copper sulphate pentrahydrat in 8% (*v*/*v*) phosphoric acid solution, which were fluorescent. Lipid bands were developed at 120 °C in 20 min and visualized and quantified by a Kodak Image Station E440CF (Kodak, Glostrup, Denmark).

### 2.9. Western Blotting

The Western blotting protocol has previously been described in detail [27]. Briefly, biopsies were sectioned on a cryostat and homogenized in ice-cold lysis buffer with protease and phosphatase inhibitors using a Bullet Blender bead-mill at 4 °C (Next Advance, Averill, NY, USA). Equal amounts of extracted muscle proteins (3 μg/μL) were separated on 4–15% or 7% (Tris-Glycine eXtended) TGX polyacrylamide gels (Bio-Rad, Hercules, CA, USA) at 200 V for 30 min. Proteins were transferred to PVDF membranes at 1.25 A for 5 min using a Trans-Blot Turbo (Bio-Rad) and blocked in Bailey’s Irish Cream (Dublin, Ireland) for 30 min and washed in TBS-T to remove excess Bailey’s. Membranes were incubated overnight with antibody against porin (#456500; Thermo Fisher Scientific, Waltham, MA, USA). As a loading control, anti-α-tubulin was used at 1:100,000 (clone 12G10, DSHB). Secondary antibodies coupled with horseradish peroxidase diluted 1:10,000 were used to detect primary antibodies (DAKO, Glostrup, Denmark). Immunoreactive bands were detected using a SuperSignal West Dura kit (Thermo Fisher Scientific, Waltham, MA, USA), quantified using a GBox XT16 darkroom (Syngene, Cambridge, UK), and GeneTools software version 4.7 (Syngene, Cambridge, UK) was used to measure the intensities of immunoreactive bands on 16-bit digital photos. Immunoreactive band intensities were normalized to the intensity of the α-tubulin for each subject to correct for differences in total muscle protein loaded on the gel.

### 2.10. Morphological Measurements

Muscle fiber morphology was evaluated by histological staining of 10 mm frozen serial sections with modified Gomori trichrome, cytochrome oxidase (COX), succinic dehydrogenase (SDH), cytochrome oxidase/succinic dehydrogenase (COX/SDH), haematoxylin and eosin (H&E), and ATPase at pH 4.3 and 10.3. Entire sections (500–3500 fibers) were evaluated for all variables, except muscle fiber size analyses (140–220 fibers) that required a perfect cross-section orientation of the biopsy. A computer imaging analysis software was used for the assessment of muscle fiber type and size (Tema, CheckVision, Hadsund, Denmark), while all other stains were evaluated with a microscope (Nikon, Tokyo, Japan) at 10–40× magnifications. To ensure that included subjects did not have a myopathy including mitochondrial myopathy, muscle sections were stained with trichrome COX, SDH, and COX/SDH in order to identify ragged red or blue fibers and to evaluate number of fibers with centrally located nuclei, which indicates an excessive turnover, if the level is over 2%. To evaluate the training response on fiber type and size, muscle sections were stained with ATPase stain [21].

### 2.11. Statistics

Data are presented as mean ± SEM, except for subject characteristics, which are given as mean ± SD. All statistical analyses were performed in Sigmaplot v11.0 (Systat software, San Jose, CA, USA), and data were evaluated using one-way repeated measures analysis of variance (ANOVA) comparing dependent groups (measurements before and after training and after deconditioning). Tukeys post hoc test was performed when the ANOVA revealed significant interactions between variables. For all invasive measurements, the untrained control leg was further compared to the trained leg after the training intervention by an additional Student’s *t*-test. Differences between groups were considered statistically significant if *p* < 0.05.

## 3. Results

### 3.1. Subject Characteristics and Performance Data

Subject characteristics are given in Table 1. The subjects completed 100% of the 24 training sessions, 96% of which were completed within the planned six-week training program.

**Figure 2:**
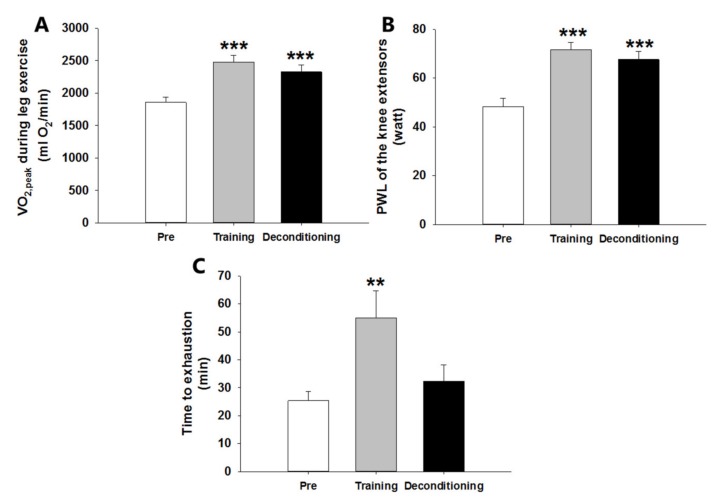
Peak oxygen uptake (VO_2,peak_) (**A**) and peak work load (PWL) (**B**) during an incremental one-legged knee-extensor exercise test and endurance exercise capacity measured as time to exhaustion in a submaximal one-legged knee-extensor endurance exercise test (**C**) before (Pre) and after six weeks of one-legged knee-extensor exercise endurance training (Training) as well as after four weeks of sedentary deconditioning (Deconditioning). Values are mean ± SEM. ** and *** significantly different (*p* < 0.01/*p* < 0.001) from Pre. *n* = 10 in (**A**) and (**B**). *n* = 6 in Pre group, n = 8 in Training group, and *n* = 9 in Deconditioning group due to the later introduction of this test in the continuous recruitment, training, and testing of subjects.

VO_2,peak_ during knee-extensor exercise increased by 33.4% from 1855 ± 83 ml∙min^−1^ to 2475 ± 101 ml∙min^−1^ after training compared to pretraining and remained elevated at 2324 ml∙min^−1^ ± 110 after four weeks of deconditioning (*p* < 0.001; Figure 2A). PWL of the knee extensors increased 48.6% from 48.2 ± 3.5 watts to 71.7 ± 2.9 watts after training compared to pretraining, and remained elevated after deconditioning at 67.6 ± 3.4 watts (*p* < 0.001; Figure 2B). The endurance capacity measured as time to exhaustion during knee-extensor exercise at 80% of PWL increased by 120% from 25.3 ± 3.4 min to 55 ± 9.6 min after training compared to pretraining (*p* < 0.01), and returned to pretraining level after deconditioning (Figure 2C).

### 3.2. Mitochondrial Enzyme Complex Activities

**Figure 3:**
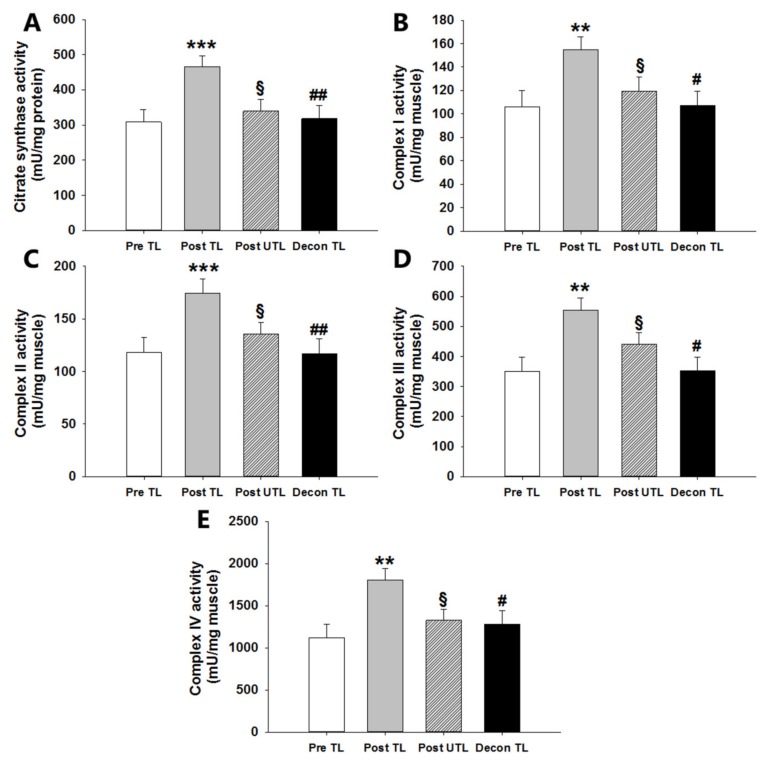
Maximal enzyme activities of citrate synthase (CS) (**A**) and mitochondrial complex I (**B**), II (**C**), III (**D**), and IV (**E**) in the exercise-trained leg before (Pre TL), in the exercised and non-exercise-trained leg after six weeks of one-legged knee-extensor exercise endurance training (Post TL and Post UTL, respectively), and in the previously trained leg after four weeks of subsequent sedentary deconditioning (Decon TL). Values are mean ± SEM. ** and *** significantly different (*p* < 0.01 and *p* < 0.001) from Pre TL. § significantly different (*p* < 0.05) from Post TL. # and ## significantly different (*p* < 0.05 and *p* < 0.01) from Post TL. *n* = 10 in Pre TL, POST TL, and Post UTL groups. *n* = 7 in Deconditioning groups due to the later introduction of this test in the continuous recruitment, training, and testing of subjects.

Maximal CS activity increased by 50.8% in the trained leg from 308 ± 35 mU/mg protein pretraining to 465 ± 31 mU/mg protein after training (*p* < 0.001) and was higher after training in the trained vs untrained leg (*p* < 0.05; Figure 3A). After deconditioning, maximal CS decreased by 31.7% to pretraining level (318 ± 38 mU/mg protein) (*p* < 0.01; Figure 3A). In addition, the maximal enzyme activity of the respiratory chain complexes all increased such that complex I through IV increased by 46% (*p* < 0.01; Figure 3B), 48% (*p* < 0.01; Figure 3C), 58% (*p* < 0.01; Figure 3D), and 61% (*p* < 0.01; Figure 3E), respectively, and the complex activities were all higher in the trained leg compared to untrained leg after training (*p* < 0.05). After deconditioning, maximal activities in complex I through IV in the previously trained leg decreased by 31% (*p* < 0.05; Figure 3B), 33% (*p* < 0.01; Figure 3C), 36% (*p* < 0.05; Figure 3D), and 28.9% (*p* < 0.05; Figure 3E), respectively, and were after deconditioning not different from pretraining levels.

### 3.3. mtDNA Content

**Figure 4:**
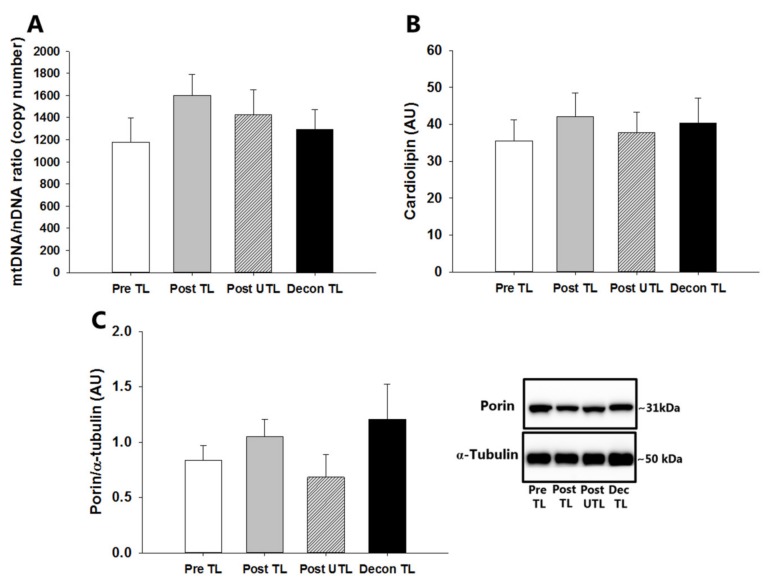
Mitochondrial DNA/nuclear DNA ratio (mtDNA/nDNA ratio (**A**), cardiolipin (**B**), and porin protein content with representative Western blots to the right (**C**) in the exercise-trained leg before (Pre TL), in the exercised and non-exercise-trained leg after six weeks of one-legged knee-extensor exercise endurance training (Post TL and Post UTL, respectively), and in the previously trained leg after four weeks of subsequent sedentary deconditioning (Decon TL). Values are mean ± SEM. *n* = 10 in Pre TL, POST TL, and Post UTL groups. *n* = 7 in Deconditioning groups due to the later introduction of this test in the continuous recruitment, training, and testing of subjects. AU; arbitrary units.

mtDNA content in skeletal muscle did not change in response to aerobic training in the trained leg and was also unaffected by subsequent deconditioning (Figure 4A). Thus, mtDNA content was also similar in the trained compared to the untrained leg after the training period. 

### 3.4. Cardiolipin and Porin Contents

Cardiolipin (Figure 4B) and porin (Figure 4C) contents in skeletal muscle did not change in response to training and were also unaffected by subsequent deconditioning. Thus, cardiolipin and porin contents were also similar in the trained compared to the untrained leg after the training period.

### 3.5. Muscle Morphology

**Figure 5:**
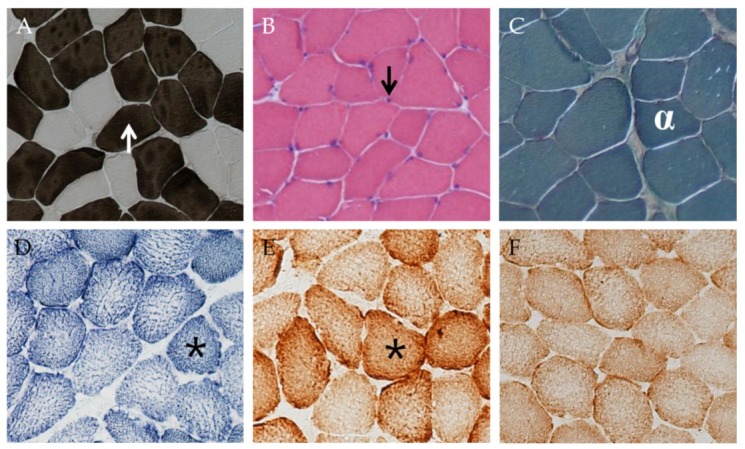
Sections of the lateral vastus muscle in the same subject after training stained for (**A**) adenosine triphosphatase (ATPase) pH 4.3, (**B**) haematoxylin & eosin (H&E), (**C**) trichrome, (**D**) succinic dehydrogenase (SDH), (**E**) cytochrome oxidase, and (**F**) COX/SDH. The white arrow (↑) shows the darkly stained type I fibers of the ATPase pH 4.3 stain. The black arrow (↓) shows myonuclei stained with H&E. White alpha (α) shows the darkly stained type I fiber (containing many mitochondria) with the trichrome stain. Black stars (*) show the darkly stained type I (high content of SDH and COX enzymes) with the SDH and COX stains. In (**F**), fibers devoid of cytochrome oxidase activity would appear blue. The ATPase stains showed that fiber type distribution and size did not change with training or detraining. There were few examples of regenerating cells, as indicated by centrally located nuclei, and no inflammation seen with the H&E staining. Staining with SDH, COX, COX/SDH, and trichrome showed no abnormalities either prior to or after training, indicating no mitochondrial dysfunction. Notice in (**D**) and (**E**) how the peripheral circumference of the type I fibers in some places stains significantly darker than central areas. This indicates a higher density of clustered subsarcolemmal mitochondria relative to the intermyofibrillar mitochondria.

Pretraining, subjects did not have indices of mitochondrial disease or other muscle disease with <1% COX-negative or ragged-red fibers, no abnormal mitochondria or central nuclei on HE staining, and no fat infiltration, connective tissue or inflammation. Training did not change this, and there was no difference in muscle biopsy findings histologically between the trained and untrained leg in regards to fiber type distribution or fiber size (Table 2; Figure 5).

## 4. Discussion

The “gene dosage theory” expresses that mtDNA replication is an integral mechanism for exercise-induced adaptations in mitochondrial enzymatic activity and function [20], which indicates that mtDNA and mitochondrial enzyme activities change proportionally [9,17,18]. However, findings of unchanged mtDNA content despite substantial changes in mitochondrial volume and enzyme activity have challenged this theory [21,22,23,24]. In order to test this, we specifically investigated mtDNA content in muscle after six weeks of one-legged, knee-extensor exercise training and four weeks of deconditioning. To make sure that changes found in the trained leg were induced by the training intervention, findings in the trained leg were compared to the contralateral, untrained leg. The study showed that six weeks of one-legged endurance exercise induced a significant training response evidenced by an enhanced maximal power output and improved oxygen uptake and endurance performance in accordance with previous observations [28,29,30,31,32]. This was accompanied by an induction of mitochondrial enzymatic activity signified by an increased maximal activity of citrate synthase (CS) and the mitochondrial respiratory chain complexes in the skeletal muscle of the exercise-trained leg, but not in the untrained leg, congruent to previous literature [28,33,34]. Conversely, four weeks of deconditioning reduced endurance capacity and mitochondrial enzymatic activity as evidenced by a decrease in CS and mitochondrial respiratory chain complex activities in the previously trained leg. Despite these substantial changes in mitochondrial function with training and deconditioning, the relative mtDNA content and mitochondrial membrane markers, porin and cardiolipin, remained unchanged. These findings emphasize that exercise-training- and deconditioning-induced changes in mitochondrial enzymatic activity is independent of genomic dosage.

It is well established that endurance exercise training increases mitochondrial function, enzyme expression, and density [35,36,37,38]. MtDNA content has repeatedly been used as an index of mitochondrial function and content, since the assumption has been that there is a 1:1 adaptability to exercise of mitochondrial content and mtDNA level in skeletal muscle. This notion has been based on findings of a similar increase in biomarkers of mitochondrial content and function and mtDNA copy number in studies performed in rodents [13,14,15] and humans [9,16,17,18,19] after 2–24 weeks of endurance training. The unaltered mtDNA content in skeletal muscle after six weeks of aerobic exercise training obtained in the present study, despite a considerable increase in other markers of mitochondrial function and enzymatic activity, questions this assumption. The findings in the present study are supported by previous studies of unaltered mtDNA content and associated large increases in mitochondrial enzyme activity in human skeletal muscle after 2−12 weeks of exercise training [21,22,23,24] and could suggest that very prolonged and intensive exercise training interventions are needed to observe changes in mtDNA content. Interestingly, studies investigating the effect of an acute bout of exercise on mtDNA content have shown a decreased mtDNA content, without altering the mitochondrial protein content and biomarkers of mitochondrial volume [10,23,39] supporting a dissociation between mtDNA and mitochondrial function in human skeletal muscle. In addition, a study investigating mitochondrial volume in skeletal muscle in sedentary subjects did not find correlation between mtDNA content and mitochondrial volume measured by electron microscopy [40], further indicating that mtDNA content is a poor biomarker of mitochondrial volume. When considering that it is well established that expression of metabolic proteins occur concomitant with an unchanged content of nuclear DNA [19,23,41], the mechanism behind the relationship between mtDNA and mitochondrial enzymatic machinery may for the same reason not be 1:1. Together, these findings underline that mtDNA content might not be a valid marker of mitochondrial adaptions to exercise training. The mechanism underlying the training-induced increase in mitochondrial enzyme levels, despite an unchanged level of mtDNA content, likely relates to increased transcription or alternatively effectiveness of post-transcriptional control, or increased translational efficiency in order to achieve balance between energy production and expenditure, when the metabolic demand is increased. 

An advantage of the present study is the comparison between a trained and an untrained muscle group in the same individual. This ensured that: (1) changes in mitochondrial enzyme activities, membrane markers, and mtDNA level were induced by the training intervention alone, (2) the biological and natural course of mtDNA content in skeletal muscle was evaluated, and (3) data variance was minimised by avoiding interpersonal differences in mtDNA content between samples. Studies have shown that mtDNA content in skeletal muscle differs between subjects despite the same training status [42,43], which was also found to be the case in the present study. Thus, by comparing an untrained with the trained leg both before and after a training intervention and again after deconditioning, we propose that we have eliminated most of the biological natural course of mtDNA content and sample variations. The methodological difference between the present and previous studies may very well be the reason why some studies have found changes in mtDNA content with training intervention. 

The present study is the first to evaluate mtDNA dynamics in human skeletal muscle after a deconditioning period. Deconditioning after exercise training has in a few studies been shown to reduce oxidative capacity and mitochondrial protein content and maximal enzyme activities in the skeletal muscle to baseline, pretraining levels after 6–8 weeks [21,29,44,45]. The present study shows that despite a substantial decrease in mitochondrial function and biogenesis judged by CS and mitochondrial complex activities, which returned to pretraining levels after four weeks of deconditioning, mtDNA content remained unchanged. The present finding underscores that even impaired mitochondrial function and protein content change independently of mtDNA content in human skeletal muscle. 

It is an ongoing debate whether training-induced changes in mitochondrial volume results in increased size or number of mitochondria or both. In the present study, mitochondrial membrane surface area and hence volume were evaluated by porin and cardiolipin content in muscle tissue. Porin, also known as voltage-dependent anion-selective channel (VDAC), is the most abundant protein of the mitochondrial outer membrane, and porin content has hence been used as a marker of total mitochondrial content [46]. To our knowledge, the present study is the first to measure porin content in human skeletal muscle in response to exercise training. The unchanged porin content after six weeks of exercise training and four weeks of deconditioning indicates that despite a substantial change in mitochondrial enzyme activities with exercise training and deconditioning, mitochondrial outer membrane area did not change accordingly. Cardiolipin, which is a phospholipid located in the inner mitochondrial membrane, is suggested to stabilize the complexes in the electron transport chain [47] and found to be a good biomarker of the total mitochondrial cristae surface area in human skeletal muscle [40]. Only a few studies have previously investigated training effects on cardiolipin content in human skeletal muscle [3,4,48]. In line with our findings, recently published data from Nielsen et al. found that 10 weeks of aerobic exercise training did not change mitochondrial cristae density in sedentary obese individuals both with and without type 2 diabetes [49]. In contrast, other studies found that a longer training period of 16 weeks of aerobic training in obese subjects with type 2 diabetes increased cardiolipin content by 55–60% in skeletal muscle [3,4]. Also, despite no effect of 10 weeks of exercise training on cristae density in Nielsen et al. [49], cross-sectional findings in the same study showed increased content of mitochondrial inner membrane surface per mitochondrial volume as a measure of cristae density in skeletal muscle of trained compared to sedentary subjects. Collectively, this indicates that mitochondrial cristae density seems to adapt to training, but that longer training interventions (>10 weeks) may be needed to detect this. This is likely also the case for the effect of training on mitochondrial number. Thus, in a recent study, six weeks of training increased mitochondrial volume, which was suggested to be driven by mitochondrial enlargement rather than synthesis of more mitochondria [50]. The authors argued that the relationship between number of mitochondrial profiles within a given area of the skeletal muscle sections and length of the mitochondrial network underscored that mitochondrial volume was increased without changes in mtDNA content or mitochondria number [50]. Collectively, this supports the notion that de novo biogenesis of new mitochondria may be enhanced with time, but the fast induction of changes in enzyme activity that are seen even after a few days or weeks of endurance exercise training in the present study and previously [23,51] does not translate into increased mitochondrial number. Instead, increases in mitochondrial number seem to be independent of this, but mitochondrial number may increase after months of intense endurance training.

Instead of using mtDNA content as an indicator of mitochondrial function in skeletal muscle, maximal mitochondrial enzyme activity appears to be a more reliable marker of mitochondrial function in adaption to exercise training in human skeletal muscle. Thus, the increase in oxidative capacity of the muscle measured as VO_2,peak_ was associated with an increase in CS and mitochondrial complex activities with exercise training in the present study. This is substantiated by findings of maximal CS and complex I activities strongly correlated with mitochondrial volume measured by electron microscopy in skeletal muscles of healthy, young men [40]. Moreover, maximal CS activity correlated with the enhancement of mitochondrial volume in response to six weeks of endurance training in human skeletal muscle [50]. Together, this points at maximal CS activity being a suitable and valid surrogate to determine exercise-training-induced changes in mitochondrial function and/or volume, but not the number of mitochondria.

Peroxisome proliferator-activated receptor gamma coactivator 1-alpha (PGC−1α) in skeletal muscle is activated and increased in expression during and after acute exercise and known to regulate mitochondrial function and content and hence could be a marker of mitochondrial adaptations to exercise training [52]. However, despite numerous attempts, we were not able to verify specific and valid antibodies or measure PGC−1α in a detectable degree for immunoblotting of PGC−1α protein content in human skeletal muscle in the present study.

It can be argued that the range in BMI of the subjects may have an impact on the overall mitochondrial function. However, that relationship is mainly due to mitochondrial activity in subcutaneous adipocytes [53]. In addition, a previous study has reported that muscle mitochondrial respiratory capacity and content were normal in younger obese humans [54] and obese humans are found to be able to induce similar mitochondrial adaptions to exercise training as lean, normal-weighed subjects [2,55,56,57]. Hence, since findings were not only compared among subjects, but also in trained and untrained muscle groups in the same individual, the impact of differences in BMI among subjects on findings were limited.

The design of this controlled intervention study, with numerous in-depth mechanistic analyses of muscle mitochondria and molecular metabolism, limited the number of study participants. However, the number of subjects included in the study was higher than indicated by our initial power calculations. Also, we would like to emphasize that we included more subjects in the present study than most other training studies, compliance was ensured by using supervised training, and findings were compared not only among subjects of the same age and gender, but also compared between a trained and an untrained muscle group in the same individual.

In the present study, only healthy young males were included. This was chosen since studies have indicated that there might be a substantial gender-associated difference in mitochondrial volume per se and moreover, studies have indicated that training effect might be different in men than that found in women. Thus, in order to avoid gender-associated changes, we decided to include only one sex. By this, we were also able to avoid the gender-associated difference when comparing results from other studies, since most other training studies merely have used young, healthy men. Future mechanistic studies are needed to explore whether the mitochondrial response to exercise training and deconditioning is influenced by gender, including hormonal status (pre- and postmenopausal women). Thus, the present study only indicates training-induced differences in young, healthy men.

In summary, the present study indicates that in healthy, young men, the adaptations in mitochondrial enzymatic activity after six weeks of aerobic endurance training and four weeks of deconditioning are independent of changes in mitochondrial genome level.

## Figures and Tables

**Table 1:** Subject characteristics at baseline.

Subject Characteristics	Mean ± SD	Range
Age, yr	22.7 ± 2.8	(18–27)
Body mass, kg	74.2 ± 15.7	(60–107)
Body fat, %	20.1 ± 7.2	(10.8–37.2)
BMI, kg∙m^−2^	22 ± 4.2	(18.3–32.7)
VO_2,max_, ml·kg BM^−1^·min^−1^	46.8 ± 6.8	(34.4–53.4)

Values are mean ± SD. BM, body mass; BMI, body mass index (weight/height^2^); VO_2,max_, maximal oxygen consumption rate; yr, years.

**Table 2:** Morphological data in the exercise-trained leg before (Pre TL), in the exercised and non-exercise-trained leg after six weeks of one-legged knee-extensor exercise endurance training (Post TL and Post UTL, respectively), and in the previously trained leg after four weeks of subsequent sedentary deconditioning (Decon TL).

Morphological Data	PRE TL	POST TL	Post UTL	Decon TL
Fiber type I, %	38.79 ± 5.32	43 ± 5.32	47.65 ± 5.59	35.72 ± 3.27
Fiber type II %	59.21 ± 5.32	57 ± 5.32	52.35 ± 5.59	64.28 ± 3.27
Fiber size type I, μm^2^	3432 ± 739	4652 ± 556	4093 ± 262	4572 ± 741
Fiber size type II, μm^2^	3562 ± 669	4875 ± 669	4998 ± 523	4946 ± 414
Central nuclei, %	0.65 ± 1.11	0.25 ± 1.11	0.68 ± 2.02	1.44 ± 1.84

Values are mean ± SEM. *n* = 8 in Pre TL, POST TL, and Post UTL groups. *n* = 4 in Deconditioning groups due to the later introduction of this test in the continuous recruitment, training, and testing of subjects.
